# Release of Full-Length PrP^C^ from Cultured Neurons Following Neurotoxic Challenge

**DOI:** 10.3389/fneur.2012.00147

**Published:** 2012-10-22

**Authors:** Kevin K. W. Wang, J. Susie Zoltewicz, Allen Chiu, Zhiqun Zhang, Richard Rubenstein

**Affiliations:** ^1^Departments of Psychiatry and Neuroscience, Center of Neuroproteomics and Biomarker Research, McKnight Brain Institute, University of FloridaGainesville, FL, USA; ^2^Laboratory of Neurodegenerative Diseases and Central Nervous System Biomarkers, Departments of Neurology and Physiology/Pharmacology, State University of NewYork Downstate Medical CenterBrooklyn, NY, USA; ^3^Banyan Laboratories, Banyan Biomarkers Inc.Alachua, FL, USA

**Keywords:** cellular prion protein, neurotoxins, maitotoxin, NMDA, Calpain, rat cerebrocortical neurons

## Abstract

The susceptibility of the normal cellular prion protein isoform, cellular prion protein (PrP^C^), to proteolytic digestion has been well documented. In addition, a link between PrP^C^ and the cytosolic protease, calpain, has been reported although the specifics of the interaction remain unclear. We performed *in vitro* and in cell-based studies to examine this relationship. We observed that human recombinant PrP (HrPrP) was readily cleaved by calpain-1 and -2, and we have identified and defined the targeted cleavage sites. In contrast, HrPrP was resistant to caspase-3 digestion. Unexpectedly, when brain lysates from PrP^C^-expressing mice were treated with calpain, no appreciable loss of the intact PrP^C^, nor the appearance of PrP^C^ breakdown products (BDPs) were observed, even though alpha II-spectrin was converted to its signature calpain-induced BDPs. In addition, when rat cerebrocortical neuronal cultures (RtCNC) were subjected to the two neurotoxins at subacute levels, maitotoxin (MTX) and *N*-methyl-d-aspartate (NMDA), PrP^C^-BDPs were also not detectable. However, a novel finding from these cell-based studies is that apparently full-length, mature PrP^C^ is released into culture media from RtCNC challenged with subacute doses of MTX and NMDA. Calpain inhibitor SNJ-1945 and caspase inhibitor IDN-6556 did not attenuate the release of PrP^C^. Similarly, the lysosomal protease inhibitor, NH_4_Cl, and the proteasome inhibitor, lactacystin, did not significantly alter the integrity of PrP^C^ or its release from the RtCNC. In conclusion, rat neuronal PrP^C^ is not a significant target for proteolytic modifications during MTX and NMDA neurotoxic challenges. However, the robust neurotoxin-mediated release of full-length PrP^C^ into the cell culture media suggests an unidentified neuroprotective mechanism for PrP^C^.

## Introduction

The cellular prion protein (PrP^C^) is a host-coded, glycosylphosphatidylinositol (GPI)-anchored (linked through Serine-231) transmembrane glycoprotein found in all mammalian cells with relatively high levels in the central nervous system (CNS; Kretzschmar et al., 1986; Moudjou et al., 2001) particularly at neuronal synapses (Sales et al., 1998). Following prion agent infection, the protease-sensitive PrP^C^ is converted into the conformationally altered, disease-specific PrP^Sc^ isoform which is partially protease-resistant (Riesner, 2003). In contrast to PrP^Sc^, PrP^C^ is soluble in detergents and sensitive to proteolytic digestion by PK.

Although PrP^C^ has been associated with numerous cellular roles associated with cell signaling and/or neuroprotective functions, the specific physiologic function of PrP^C^ remains unclear. PrP^C^ neuroprotective functions that have been demonstrated both *in vivo* and *in vitro* (Westergard et al., 2007; Linden et al., 2008) include: cytoprotective activity against internal or environmental stresses that initiate apoptosis and oxidative stress, ionic channel modulation, transmembrane signaling, as well as formation and maintenance of synapses. PrP^C^ not only protects neurons *in vitro* and *in vivo* from *N*-methyl-d-aspartate (NMDA)-evoked excitotoxicity (Khosravani et al., 2008) but also against oxidative stress and plays a role in Cu/Zn superoxide dismutase activity (Brown and Besinger, 1998; Wong et al., 2000; Brown et al., 2002).

PrP^C^ and PrP^Sc^ are subject to diverse intracellular proteolytic processing events (Pan et al., 1992; Harris et al., 1993; Taraboulos et al., 1995). PrP^C^ undergoes proteolytic cleavage at amino acids 110/111 within a segment of conserved hydrophobic amino acids to produce a ∼17 kDa C-terminal fragment referred to as C1. Studies suggest that ADAM/TACE (a disintegrin and metalloprotease/tumor necrosis factor α-converting enzyme) matrix metalloproteases may be responsible for the generation of the C1 fragment (Vincent et al., 2001). Previous studies using post-mortem human brain extracts demonstrated that the disease-associated PrP in Creutzfeldt–Jakob disease (CJD) brains is cleaved by a cellular protease to generate a C-terminal fragment, referred to as C2, which has the same molecular weight as PrP27–30, the protease-resistant core of PrP^Sc^ (Chen et al., 1995). Dron et al. (2010) showed that the full-length PrP^Sc^ to C2 ratio varied such that uncleaved PrP^Sc^ accumulated in primary neurons and brain whereas PrP^Sc^ processing occurred in infected Rov and MovS cells. They also found that cathepsin, but not calpain, inhibitors markedly reduced C2 formation. In contrast, studies by Yadavalli et al. (2004) using persistently infected scrapie mouse brain cells or persistently infected scrapie N2a cells indicate that endoproteolytic cleavage of PrP^Sc^ is facilitated by calpains.

Wang et al. (2005) showed that in several cell lines and in primary mouse cortical neurons PrP^C^ was normally degraded by the proteasome but could alternatively be targeted by calpain. Furthermore, Hachiya et al. (2011) reported that the calpain inhibitor, calpastatin, dramatically inhibited normal endoproteolysis of PrP^C^ in N2a cells. Also, they found that the molecular weight of PrP^C^ fragments generated by spontaneous proteolysis was identical to those produced when PrP^C^ translated *in vitro* was exposed to exogenously added calpain. Due to these conflicting data regarding the potential vulnerability of PrP^C^ to calpain and other cytosolic proteases, we performed *in vitro* and in cell-based studies using rat primary cerebrocortical neuronal cultures (RtCNC). We describe the possibility of a novel neuroprotective function for calpain-resistant, full-length PrP^C^ that is shed from neuronal cells in response to neurotoxic challenge.

## Materials and Methods

### Materials

Secondary antibodies were purchased from Novagen (Philadelphia, PA, USA). Maitotoxin (MTX) and clasto-lactacystin-β-lactone from Calbiochem (Billerica, MA, USA). NMDA, NH_4_Cl, and other chemical reagents were from Sigma-Aldrich (St. Louis, MO, USA). Human recombinant calpain-1, rat calpain-2, and human recombinant caspase-3 were from EMD Millipore Biosciences (Billerica, MA, USA). Calpain inhibitor SNJ-1945 (SNJ) was a gift from Senju Pharmaceutical (Shimazawa et al., 2010) while pan caspase inhibitor IDN-6556 (IDN) was synthesized in-house at Banyan Laboratories according to published methods (Hoglen et al., 2004). Cell culture grade reagents were from Invitrogen (Grand Island, NY, USA). Anti-alpha II (αII)-spectrin monoclonal antibody (Mab) was purchased from Enzo Life Sciences (Farmingdale, NY, USA) while anti-PrP Mabs 7E4, E11, and D8 were generated by Dr. Rubenstein at SUNY Downstate Medical Center. Since rat PrP is not commercially available. We used human recombinant PrP (HrPrP) as a substitute. The human PrP (accession # AAH22532) and rat PrP (accession # BAA08790) show a high level of homology. Purified *E. coli* HrPrP (residues 23–231 based on the human PrP sequence mimicked signal peptidase removal of the first 22 residues) containing an N-terminal (His)_6_-tag fusion protein, designated (His)_6_-HrPrP, was purchased from Abnova (Walnut, CA, USA) and EMD Millipore Bioscience (Billerica, MA, USA).

### RtCNC preparation and neurotoxin challenges

The Institutional Animal Care and Use Committee at the University of Florida (Gainesville, FL, USA) approved the use of timed pregnant rats for these studies and all animal use followed the appropriate regulatory standards. RtCNC were prepared from fetal Sprague Dawley rats as previously described (Wang et al., 1996). These neuronal-enriched cultures were maintained *in vitro* and allowed to mature for 10 days. Cells were then either untreated (control), exposed to dimethylsulfoxide (DMSO) vehicle alone, or challenged with neurotoxins (0.3 nM MTX or 300 μM NMDA) for 24 h in the absence or presence of lysosomal neutralizing agent NH_4_Cl (10 mM), proteasome inhibitor lactacystin (10 μM), SNJ (30 μM), or IDN (30 μM). Inhibitors were added 1 h prior to addition of neurotoxic drug. All treatments were performed in serum-free Dulbecco’s Minimal Essential Medium in a volume of 300 μl per well of a 12-well cell culture plate. DMSO vehicle alone as well as all drug treatments had no adverse affects on cell morphology and viability for at least 72 h (data not shown). After treatments, conditioned media was collected from each sample into separate tubes on ice and clarified by microcentrifugation at 10,000 × *g* for 5 min. Supernatants (“conditioned cell culture media”) were immediately frozen at −80°C.

Soluble cell extracts were generated by gently shaking cells for 2 h at 4°C in 1x Triton X-100 lysis buffer (20 mM Tris-HCl, pH 7.4, 150 mM NaCl, 5 mM EDTA, 5 mM EGTA, 1% Triton X-100, 1 mM dithiothreitol (DTT), 1x Roche complete protease inhibitor cocktail, 1x phosphatase inhibitors (Sigma). This treatment resulted in complete cell lysis by microscopic observations. Extracts were transferred to 1.5 ml microcentrifuge tubes, spun at 10,000 × G for 10 min at 4°C to generate insoluble pellets, and both were stored at −80°C. The insoluble pellets were then extracted and resuspended in 1x RIPA buffer (50 mM Tris-HCl, pH 8.0, 150 mM NaCl, 5 mM EDTA, 0.5% Igepal CA-630, 0.5% sodium deoxycholate, 0.2% SDS) supplemented with 1x protease inhibitor cocktail and disrupted by cup horn sonication (Sonics Vibracell, Newtown, CT, USA) for three 10 s. on−10 s. off cycles (20% power output). The samples were microcentrifuged at 10,000 × G for 10 min at 4°C and the supernatants, labeled as insoluble cell extracts, were stored at −80°C. Unless otherwise stated, 50 μg each of soluble and insoluble fractions and 20 μl conditioned media were analyzed by SDS-polyacrylamide gel electrophoresis (SDS-PAGE) and western blotting.

For Endoglycosidase H (Endo H) treatment, combined 25 μl of samples (soluble or insoluble cell extracts, conditioned cell culture media) with 3 μl of 10x glycoprotein denaturing buffer (5% SDS, 0.4 M DTT) and 2 μl of distilled water. Boiled for 10 min to denature sample and allowed to cool. Next, added a final concentration of 0.05 M sodium citrate (pH 5.5) and 500 units of Endo H (New England BioLabs, Ipswich, MA, USA) in a total final reaction volume of 40 μl. Incubated the reaction for 2 h at 37°C and stopped by boiling for 4 min in 1x SDS-PAGE sample buffer. The samples were electrophoresed on 12% gels and immunoblotted as described below.

### Mouse brain lysate preparation

Adult naive mice were anesthetized and immediately sacrificed by decapitation. Brain was exposed and flushed with ice-cold phosphate-buffered saline (PBS). The bilateral cortex was immediately removed, rinsed with ice-cold PBS, and placed into a 1.5 ml microcentrifuge or cryofreeze tube, snap-frozen in liquid nitrogen and stored at −80°C until used. For brain lysates, samples were pulverized to a fine powder with a mortar and pestle set into dry ice. The pulverized brain tissue powder was then treated for 90 min at 4°C with 1X Triton X-100 lysis buffer. The brain lysates were then centrifuged at 8,000 × G for 5 min at 4°C and the supernatants were snap-frozen in liquid nitrogen and stored at −80°C. Protein concentrations of samples were determined using the Bio-Rad DC protein assay.

### SDS-PAGE, immunoblotting, and statistics

Brain tissue extracts (20 μg), soluble and insoluble RtCNC cell lysate fractions (50 μg), and conditioned media (20 μl) were separated by SDS-PAGE (10–20% gradient gels for HrPrP, 12% gels for PrP^C^, 4–20% gradient gels for spectrin), and transferred to PVDF membrane by iBlot (Invitrogen) or the semi-dry method. Blots were blocked in 5% non-fat dry milk in Tris-buffered saline containing 0.2% Tween-20, pH 7.4 (TBST), and then probed with primary Mabs (anti-PrP Mabs 7E4, E11, or D8 at 1 μg/ml, and anti-αII-spectrin at 0.05 μg/ml) overnight. The bands were either visualized by chemiluminescence using a goat anti-mouse IgG-horseradish peroxidase conjugate (ECL Super Signal West Dura, Pierce) or by incubation with biotin-conjugated secondary antibody followed by streptavidin-alkaline phosphatase conjugate and developed with NBT-BCIP substrate. Quantification of PrP^C^, as represented by the non-, mono-, and deglycosylated isoforms of the protein, was performed by densitometric analysis using NIH Image J software v. 1.34. In SDS-PAGE and Western blot experiments, it is noted for *in vitro* protein digestion, we loaded the same amount of starting protein concentration before digestion and made sure equal sample volume was loaded. For cell lysate studies, we routinely run Ponceau S staining and/or β-actin to ensure protein loading is the same for each lane. Unless stated otherwise, all data shown are representative of three separate experiments.

### *In vitro* proteolysis of (His)_6_-HrPrP or PrP^C^ in mouse brain lysate

A 100 μg aliquot of (His)_6_-HrPrP or mouse brain lysate was subjected to calpain-1, calpain-2, or caspase-3 digestion (each at a protease:substrate ratio of 1:50). For (His)_6_-HrPrP, following SDS-PAGE, the proteins were transferred to PVDF membrane and stained with Coomassie Brilliant Blue (0.6% wt/vol in 1:1 methanol:H_2_O) for 15 s followed by methanol:H_2_O (1:1) destaining until protein bands were visible. After soaking in water, PVDF membranes were air-dried. Major breakdown products (BDPs) were identified by N-terminal microsequencing performed at the Interdisciplinary Center for Biotechnology Research (University of Florida, Gainesville, FL, USA). Alternatively, a 5 μg aliquot of (His)_6_-HrPrP was digested with calpain-2 and 0.5 μg protein were analyzed per lane.

## Results

### Calpain processing of HrPrP and identification of major calpain cleavage sites

**Figure 1:**
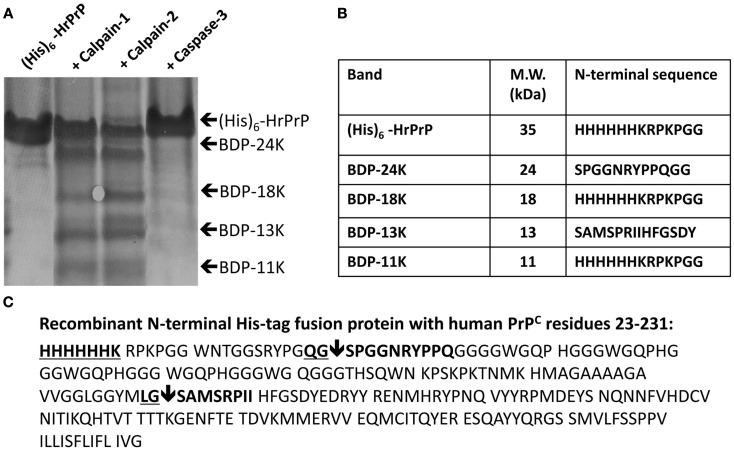
**HrPrP vulnerability to calpain-1, -2 digestion with identification of major cleavage sites**. *E. coli* expressed recombinant N-terminal (His)_6_-tag fusion protein with human PrP^C^ residues 23–231 (Human prion protein; accession # NP 898902) was subjected to calpain-1, calpain-2, and caspase-3 digestion (1:50 protease:substrate ratio). Following SDS-PAGE, proteins were transferred to PVDF membrane and stained with Coomassie blue. **(A)** Major BDPs were identified by N-terminal microsequencing. **(B)** Based on the PrP^C^ amino acid sequence the cleavage sites are identified and depicted by downward arrows. The new N-terminal sequences are identified in bold and the calpain recognition P1–P2 sequences are underlined and in bold **(C)**.

The issue of PrP^C^ vulnerability to calpain proteolysis was first addressed. The *E. coli* expressed (His)_6_-HrPrP was subjected to calpain-1, calpain-2, and caspase-3 digestion (Figure 1). Calpain-1 and calpain-2 are the two dominant isoforms of calpain in neurons. Caspase-3 treatment was also examined since it has previously been shown that many calpain substrates also serve as substrates for caspase-3 (Wang, 2000).

Following SDS-PAGE and Coomassie blue staining, intact (His)_6_-HrPrP migrated as an ∼30 kDa protein, while four major BDPs of molecular weights 24, 18, 13, 11 kDa were observed when (His)_6_-HrPrP was digested by either calpain-1 or calpain-2 (Figure 1A). In contrast, caspase-3 digestion yielded no BDPs or observable reduction of intact (His)_6_-HrPrP intensity (Figure 1A). N-terminal microsequencing of these proteins identified PrP-BDP-18 and PrP-BDP-11 kDa as N-terminal PrP products each containing an intact His-tagged N-terminus (Figure 1B). PrP-BDP-24 kDa contained a new N-terminus beginning at residue 40 (S_40_PGGNRYPPQGG), while PrP-BDP-13 kDa possessed a new N-terminus beginning at residue 131 (S_131_AMSPRIIHFGSDY; Figures 1B,C). Thus the two major calpain cleavage sites were identified as between PrP residues 40–41 and residues 130–131(Figure 1C).

**Figure 2:**
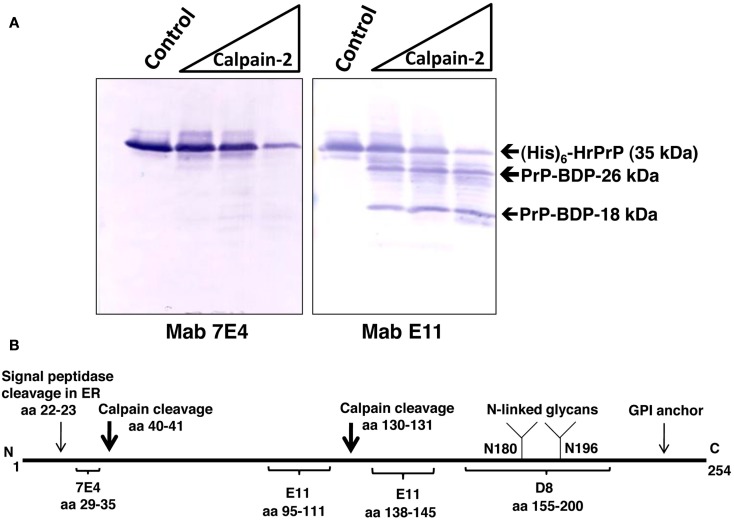
**Characterization of HrPrP proteolysis by calpain-2**. **(A)** Recombinant N-terminal (His)_6_-tag PrP fusion protein was untreated (control) digested with calpain-2 (protease:substrate ratios of 1:200, 1:50, 1:5). Five hundred nanograms of protein was then subjected to SDS-PAGE and western blotting. Three antibodies were used. Anti-PrP Mabs 7E4, E11, and anti-His tag antibody. Rainbow molecular weight markers were used. **(B)** Schematic of PrP showing the positions of the two calpain cleavage sites and the anti-PrP Mab epitopes.

Immunostaining was performed to further characterize calpain-2 induced proteolytic cleavage of (His)_6_-HrPrP (Figure 2). (His)_6_-HrPrP was digested with calpain-2 using three protease:substrate ratios of 1:200, 1:50, and 1:5 with the last condition causing the most extensive PrP hydrolysis. Treated protein samples (500 ng) were analyzed by western blotting using PrP-specific Mabs 7E4 and E11 (Figure 2A) as well as a rabbit polyclonal anti-His tag antibody. As previously reported (Chang et al., 2012) and depicted in Figure 2B, the epitope for Mab 7E4 are PrP residues 29–35. Since one of the targeted calpain cleavage sites is residues 40–41. MAb 7E4 antibody detected epitope residue 29–35 which is N-terminal to the first calpain cleavage site (between residue 40–41), it is thus truncated off as a very small fragment (less than 4 kDa) thus not readily observable on the blot (Figure 2A).

Instead, we observed a protease dose-dependent reduction of intact (His)_6_-HrPrP indicative of proteolysis (Figure 2A). Anti-His tag antibody, which detects the N-terminal (His)_6_-tag, produced essentially the same results as Mab7E4 (data not shown).

In contrast, anti-PrP Mab E11 targets two internal epitopes (aa 95–111 and aa 138–145; Figure 2B) and upon cleavage by calpain-2, two fragments of 26 and 18 kDa are readily observed in addition to the reduction of intact PrP (Figure 2A). These results further confirmed that (His)_6_-HrPrP can be readily digested by calpain-2.

### Vulnerability of endogenous brain PrP^C^ to calpain proteolysis

**Figure 3:**
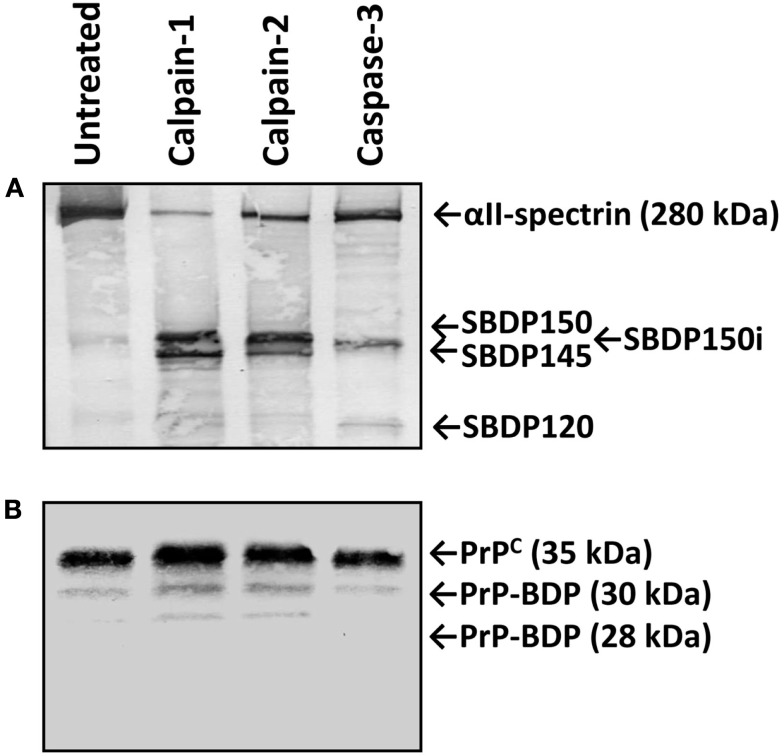
**Relative resistance of PrP^C^ from mouse brain lysate to calpain proteolysis**. Lysate from naïve mouse brains were prepared and digested with calpain-1, -2, or caspase-3 as described in section “Materials and Methods.” Aliquots of the untreated and treated lysates were then electrophoresed, western blotted, and immunostained with either anti-αII-spectrin Ab **(A)** or with anti-PrP Mab E11. **(B)** Characteristic calpain-generated SBDP150 and SBDP145 as well as caspase-3 generated SBDP150i and SBDP120 are indicated. No PrP^C^-BDPs were observed following the treatments with Mab 7E4 (data not shown).

Previous studies (Wang et al., 2005; Dron et al., 2010) have showed that PrP in different tissues or cell types might have variable vulnerability to endogenous proteolysis. Thus we next sought to examine the vulnerability of native PrP^C^ to calpain and caspase-3 proteolysis *in vitro* with mouse brain lysate under digestion conditions similar to that described for (His)_6_-HrPrP. We observed the characteristic calpain-mediated αII-spectrin BDPs (SBDPs) SBDP150 and SBDP145, while caspase-3 generated the characteristic SBDP150i and SBDP120 (Figure 3A), as has been previously described (Zhang et al., 2009). In contrast, only two very minor BDPs (30 and 28 kDa) of the 33–35 kDa PrP^C^ were observed by western blotting when immunostained with anti-PrP Mab E11 (Figure 3B). No PrP^C^-BDPs were observed following immunostaining with Mab 7E4 (data not shown). This result strongly suggests that endogenous PrP^C^, unlike (His)_6_-HrPrP, is relatively resistant to calpain proteolysis.

### Integrity and localization of PrP^C^ in RtCNC subjected to neurotoxic challenges

We next sought to examine the integrity and localization of PrP^C^ in RtCNC after they were subjected to neurotoxic challenges. Neurotoxin calcium channel opener MTX (0.3 nM) or excitotoxin glutamate analog NMDA (300 μM) were selected since it has previously been shown that these challenges induce strong calpain and mixed calpain/caspase-3 activation (Wang et al., 1996; Zhang et al., 2009).

**Figure 4:**
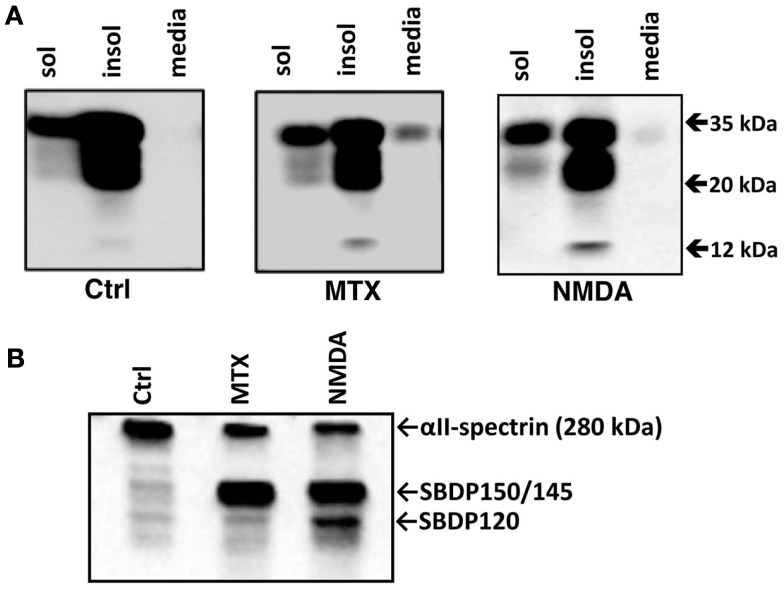
**Release of full-length PrP^C^ into culture media from RtCNC challenged with neurotoxins MTX and NMDA**. RtCNC were either untreated (control; Ctrl) or challenged with MTX or NMDA. **(A)** Soluble and insoluble cell fractions and conditioned cell culture media were analyzed by SDS-PAGE followed by western blotting and immunostaining with Mab D8. **(B)** Soluble cell fractions were also analyzed with anti-αll spectrin Mab to probe the formation of SBDPs (SBDP150 by calpain and SBDP120 by caspase-3). Blots are representative of four separate experiments.

PrP^C^ could reside in membrane-associated fractions or in soluble cytosolic fractions. We therefore performed cell extractions which allowed us to examine soluble and insoluble cellular proteins separately by initially lysing cells with mild neutral detergents followed by extraction of the remaining insoluble proteins with anionic detergents. Thus soluble and insoluble fractions (50 μg each) and conditioned cell culture media (20 μl), were analyzed by western blotting using Mab D8 (Figure 4). In the insoluble fraction of control cortical cultures we observed that the bulk of the PrP^C^ was full-length (33–35 kDa) and a protein that migrated at 20 kDa (most likely the deglycosylated form). Small amount of 33–35 kDa, and to much lesser extent the 20 kDa form, were also detected in the soluble fraction of control cultures. In contrast, there were almost undetectable levels of PrP^C^ in the conditioned cell culture media (Figure 4). MTX has previously been shown to be a potent calpain activator due to the extreme calcium influx it induces (Wang et al., 1996; Zhang et al., 2009). Yet, with MTX treatment of RtCNC, there was no significant alteration of PrP^C^ patterns in either insoluble or soluble fractions as far as the 33–35 and 20 kDa species are concerned (Figure 4A). There was, however, a minor increase of 12 kDa form (possibly a PrP^C^ BDP). Similarly, NMDA challenge did not alter the pattern of PrP^C^ in both soluble or insoluble cellular fractions with the exception that the 20 kDa protein band appeared to be slightly elevated. However, rather unexpectedly, there was a distinct presence of the intact PrP^C^ in conditioned cell culture media only with NMDA or MTX treatment, when compared to control. Given that only 20 μl of a total of 500 μl conditioned cell culture media was examined, the overall increase of PrP^C^ was relatively substantial (Figure 4A). When we added concentrated RIPA buffer to the whole cell culture well to extract and recover the insoluble, soluble fractions, and cell conditioned media with or without MTX or NMDA treatment, we observed that the overall levels of PrP^C^ remained the same, thus ruling out the increased PrP in cell media being due to overexpression and increased production of PrP^C^ by the cells (results not shown). Thus we concluded that both neurotoxin treatments in fact resulted in increased release of 33–35 kDa PrP^C^ into the media (Figure 4). Following MTX exposure, Endo H treatment of the soluble and insoluble cell extracts and the conditioned cell culture media prior to electrophoresis and western blotting did not result in either altered PrP^C^ migration or the immunostaining patterns compared to untreated controls (data not shown) suggesting that these are fully processed and mature forms of the protein. To confirm that calpain was indeed activated following MTX- and NMDA-treatments, soluble fractions were also analyzed with anti-αII-spectrin Mab to assess the formation of SBDPs. In comparison to control conditions, a major reduction of intact αII-spectrin with MTX and NMDA-treatments was observed. Furthermore, calpain-mediated SBDP150/145 bands were prominent under these neurotoxic conditions (Figure 4B).

**Figure 5:**
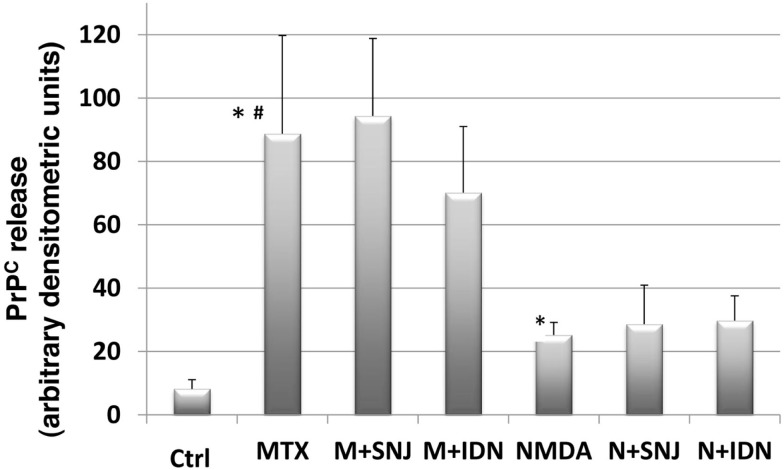
**Quantification of release of full-length PrP^C^ into culture media from RtCNC challenged with neurotoxin MTX and NMDA**. RtCNC were either untreated (control) or challenged with MTX or NMDA in the absence or presence of SNJ or IDN. Full-length PrP^C^ release was detected by analyzing conditioned cell culture media by SDS-PAGE and western blotting using anti-PrP Mab D8. Quantitation of PrP^C^ immunostaining was performed using Image J as described in the Methods section. Data are representative of up to seven measurements per group from three separate experiments. Values are expressed as mean ± standard error. * indicates NMDA or MTX challenge groups have significantly higher PrP^C^ release compared to control (*p* < 0.05). # indicates MTX challenge group has significantly higher PrP^C^ release compared to NMDA challenge group (*p* < 0.05). Neither inhibitor treatment significantly altered MTX- or NMDA-induced PrP^C^ release levels, respectively.

**Figure 6:**
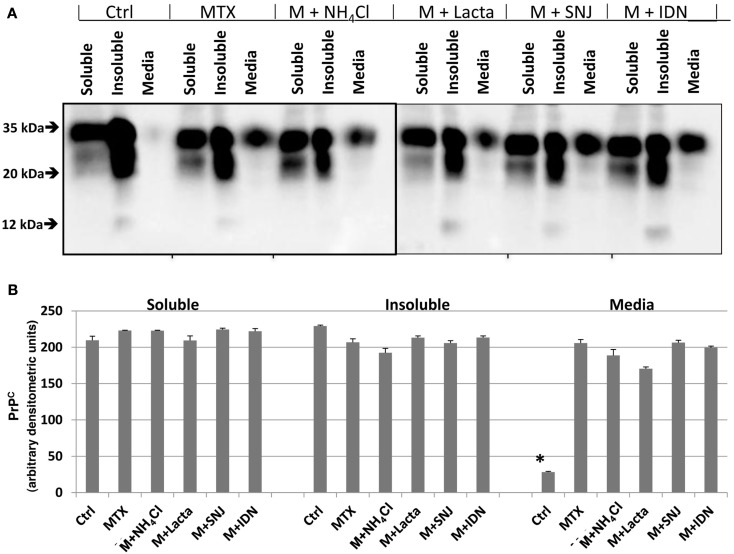
**Integrity of PrP^C^ in RtCNC and its release into culture media upon MTX challenge is not significantly altered by protease inhibition**. **(A)** RtCNC were either untreated (control) or challenged with MTX in the absence or presence of NH_4_Cl, lactacystin (lacta), SNJ, or IDN. Soluble and insoluble cell fractions and conditioned cell culture media were analyzed by SDS-PAGE and western blotting using anti-PrP Mab D8. Blots are representative of three separate experiments. (**B**) Quantification of PrP^C^ band (33–35 kDa) intensity in the fractions as described in **(A)**. Values are expressed as mean ± standard error. MTX did not alter the levels of PrP^C^ in the soluble or insoluble cell extracts. None of the protease inhibitor treatments altered PrP^C^ levels in either soluble or insoluble cell fractions. The MTX-induced PrP^C^ release into the media was not attenuated by any of the inhibitor treatments.

We examined if the unexpected release of full-length PrP^C^ induced by the neurotoxins MTX and NMDA could be attenuated by calpain or caspase-3 inhibition. RtCNC were either untreated (control) or challenged with MTX or NMDA, in the absence or presence of SNJ or IDN (Figure 5). PrP^C^ release was again monitored by immunoblotting with Mab D8. Similar to Figure 4, we confirmed that media from both MTX and NMDA challenged groups contained significantly greater PrP^C^ release compared to control (*p* < 0.05). We also noted that the MTX-induced PrP^C^ release levels were significantly higher than those by NMDA-induced release (*p* < 0.05). Neither SNJ nor IDN inhibition significantly altered MTX- or NMDA-induced PrP^C^ release levels, respectively (Figures 5 and 6).

Lastly, we investigated if the integrity of PrP^C^ or its distribution in soluble, insoluble, or media fractions could be altered by inhibition of additional cellular proteases. Here we chose the MTX challenge since it produced the most robust PrP^C^ response (Figure 5). RtCNC were either untreated (control) or challenged with MTX in the absence or presence of lysosomal neutralizing agent (10 mM NH_4_Cl), proteasome inhibitor (10 μM lactacystin), calpain inhibitor (30 μM SNJ), or caspase inhibitor (30 μM IDN). Soluble and insoluble fractions (50 μg each) and conditioned cell culture media (20 μl) were analyzed by western blotting with Mab D8. MTX did not alter PrP^C^ integrity as demonstrated by the unmodified pattern of protein banding in soluble and insoluble cell fractions (Figure 6). However, MTX treatment did cause dramatic release of full-length PrP^C^ into cell culture media, consistent with our previous findings (Figure 6). None of the protease inhibitor treatments significantly altered full-length PrP^C^ levels in either cell fractions or attenuated the MTX-induced PrP^C^ release into the media (Figure 6B). Thus, full-length PrP^C^ release from MTX-challenged cortical neurons into cell culture media appears to be independent of cellular protease activation.

## Discussion

There exists a potential vulnerability of PrP^C^ and PrP^Sc^ to processing by calpain and other cytosolic proteases (Yadavalli et al., 2004; Wang et al., 2005; Hachiya et al., 2011). However, conflicting data exists especially regarding PrP^C^. In this study, we performed *in vitro* and in cell-based studies using primary RtCNC to further examine this issue. (His)_6_-HrPrP was cleaved by calpain-1 and -2 *in vitro* and the two targeted cleavage sites were identified between resides Gly40-Ser41 and residues Gly130-Ser131. (His)_6_-HrPrP, however, was resistant to caspase-3 digestion. In contrast, PrP^C^ in brain cell lysates was fairly resistant to calpain digestion while αII-spectrin was readily converted to its signature calpain-generated BDPs. The amino acid sequences for human and rat PrP are identical at the calpain targeted HrPrP cleavage sites. Therefore, the differences in calpain sensitivity between HrPrP and the rat PrP cell cultures is probably the result of protein folding differences and target site accessibility of the recombinant protein compared to the native rat PrP^C^. Additionally, the detergent solubilization of HrPrP may contribute to calpain target site availability when compared to the more natural environment of the cell membrane-associated rat PrP^C^.

Also, when RtCNC were subjected to the MTX and NMDA neurotoxins, proteolysis of PrP^C^ was again minimal. Unexpectedly, these challenges resulted in the release of presumably full-length, mature PrP^C^ into the media. Treatment of the RtCNC with SNJ, IDN, NH_4_Cl, or lactacystin did not significantly alter the integrity of cellular PrP^C^ or its release into conditioned cell culture media.

Previous studies (Yadavalli et al., 2004) have shown that both PrP^Sc^ and PrP^C^ isoforms in CJD brain are cleaved by cellular proteases to generate a C-terminal fragment, referred to as C2 and that one of these proteases is likely to be calpain. Hachiya et al. (2011) found that the molecular weight of PrP^C^ fragments generated by spontaneous proteolysis in mouse N2a cells was identical to the calpain-generated fragments of *in vitro*-translated PrP^C^ and overexpression of the calpain inhibitor, calpastatin, drastically inhibited normal endoproteolysis of PrP^C^ in N2a cells. In addition, Wang et al. (2005) showed that in several cell lines and primary mouse cortical neurons, a portion of the endogenous PrP^C^ was degraded by the proteasome in the cytosol. Thus we initially hypothesized that PrP^C^ would be vulnerable to calpain cleavage in RtCNC and rat brain lysate but were surprised to find that was not the case. Further, data mining studies by Dron et al. (2010) reported that in primary neurons and brain tissue, PrP^Sc^ accumulated predominantly as uncleaved species. Thus, this PrP^Sc^ data is consistent with our PrP^C^ data presented here.

Taken together, we observed that PrP^C^ in RtCNC was not a significant target for proteolytic modifications during neurotoxic challenges. Yet, the current study is the first report that describes the release or shedding of full-length PrP^C^ from the cell upon neurotoxic challenges. It is possible that this is a protective response of the cells to neurotoxic threats. Importantly, the release of full-length PrP^C^ as a response to neurotoxins might have a physiological function involved in neuroprotection such as stimulating cell activation and/or release of trophic factors. It has been shown that in transgenic mice, PrP^C^ overexpression is neuroprotective while mice that did not express PrP^C^ were more susceptible to neurotoxicity or neurodegeneration (Westergard et al., 2007).

A protease-mediated physiological cleavage near the C-terminus of PrP^C^ in close proximity to its GPI-anchor has been reported in neurons and lymphoid cells (Borchelt et al., 1993). This resulted in the release of the almost full-length protein from the plasma membrane. This soluble form of shedded PrP^C^ was also found in human CSF (Tagliavini et al., 1992) and blood (Perini et al., 1996; MacGregor et al., 1999; Parizek et al., 2001) indicating physiological relevance. Cell culture experiments identified ADAM10, along with a regulatory role of ADAM9, as the active components responsible for the shedding of nearly full-length PrP^C^ from the cell surface (Cisse et al., 2005; Taylor et al., 2009; Tousseyn et al., 2009; Moss et al., 2011). *In vivo* studies also confirmed the role of ADAM10 in PrP^C^ shedding (Altmeppen et al., 2011).

As Altmeppen et al. (2012) recently articulated elegantly that when compared to membrane-associated PrP^C^, shed PrP^C^ may have distinct functions or activities (Harris et al., 1993; Parizek et al., 2001; Altmeppen et al., 2011). Amyloid precursor protein (APP) can be processed either through an amyloidogenic pathway to generate Aβ or through a non-amyloidogenic pathway to block Aβ production and generate soluble APPα (sAPPα). Aβ_1-42_ accumulation in the brain has been shown to impair neuronal function (Hu et al., 2009), while generation of sAPPα is known to play neuroprotective and neurotrophic roles (Cheng et al., 2002; Stein et al., 2004). De Felice et al. (2007) reported that Aβ oligomers caused the formation of neuronal-damaging reactive oxygen species that was mediated by NMDA receptors (NMDAR). Further, PrP^C^ has been reported to provide a neuroprotective role by its ability to limit NMDAR excitotoxicity (Khosravani et al., 2008) while both the presence of Aβ_1-42_ and PrP^C^ inactivation caused the NMDAR neurotoxicity (You et al., 2012).

Thus taken these concepts together, and relating them to neurodestructive (Aβ) and neuroprotective (sAPPα) roles of processed APP, the increased release of shed PrP^C^, could represent a self-defense mechanism to preserve those neurons or neighboring neurons under neurotoxic attacks (Altmeppen et al., 2012). Here, it is tempting to suggest that similar or same neuroprotective mechanisms might be at play in our MTX and NMDA challenge paradigm in RtCNC, thus explaining the observed PrP^C^ release. It is possible that exposure of neuronal cells to neurotoxins activates ADAM10 causing PrP^C^ shedding. This shed PrP^C^ could, in turn, bind to neurotoxins thereby reducing their direct contact with cells. Our laboratories are now in the process of furthering work in this direction.

Roberts et al. (2010) demonstrated that PrP^C^ is significantly increased in the cerebrospinal fluid of HIV-1 infected individuals with cognitive impairment. Their studies indicate that since the increase of PrP^C^ is not a generalized phenomenon of neuronal injury or neuroinflammation, it can therefore serve as a useful biomarker to monitor disease progression in HIV-infected individuals. Although the release of PrP^C^ in response to neurotoxin challenge *in vitro* is interesting and may offer some insight as to its neuroprotective mechanism, its ability to serve a similar function and/or as a CNS biomarker following brain insult is attractive but speculative and requires further investigation. The release of proteins (e.g., SBDPs, UCH-L1) from neuronal cultures subjected to neurotoxic challenges have not only been reported previously (Dutta et al., 2002; Siman et al., 2004) but have also proven to be useful CNS biomarkers after traumatic or ischemic brain injury (Zhang et al., 2011).

## Conflict of Interest Statement

The authors declare that the research was conducted in the absence of any commercial or financial relationships that could be construed as a potential conflict of interest.
